# Understanding Ionic Diffusion Mechanisms in Li_2_S Coatings for Solid-State Batteries: Development of a Tailored Reactive Force Field for Multiscale Simulations

**DOI:** 10.1021/acs.jpcc.3c04991

**Published:** 2023-11-15

**Authors:** Maddalena D’Amore, Moon Young Yang, Tridip Das, Anna Maria Ferrari, Minho M. Kim, Riccardo Rocca, Mauro Sgroi, Alessandro Fortunelli, William A. Goddard

**Affiliations:** † Dipartimento di Chimica, Università di Torino, Via P. Giuria 5, Torino 10125, Italy; ‡ Materials and Process Simulation Center (139-74), California Institute of Technology, Pasadena, California 91125, United States; ¶ Department of Chemistry, Korea Advanced Institute of Science and Technology (KAIST), Daejeon 34141, Republic of Korea; § Centro Ricerche FIAT S.C.p.A., Strada Torino 50, Orbassano, Turin 10043, Italy; ∥ CNR-ICCOM, Consiglio Nazionale delle Ricerche, via Giuseppe Moruzzi 1, Pisa 56124, Italy

## Abstract

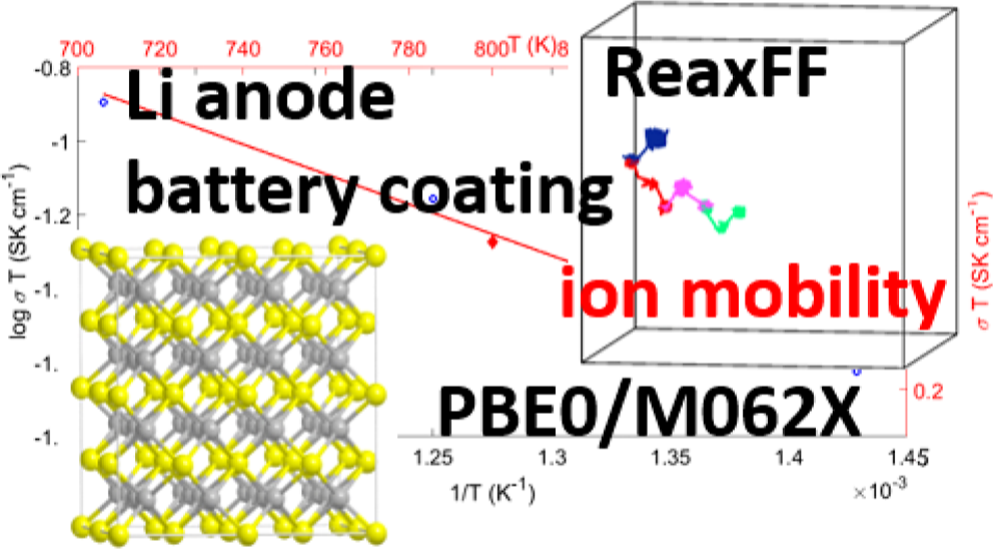

In order to investigate Li_2_S as a potential protective coating for lithium anode batteries using superionic electrolytes, we need to describe reactions and transport for systems at scales of >10,000 atoms for time scales beyond nanoseconds, which is most impractical for quantum mechanics (QM) calculations. To overcome this issue, here, we first report the development of the reactive analytical force field (ReaxFF) based on density functional theory (DFT) calculations on model systems at the PBE0/TZVP and M062X/TZVP levels. Then, we carry out reactive molecular dynamics simulations (RMD) for up to 20 ns to investigate the diffusion mechanisms in bulk Li_2_S as a function of vacancy density, determining the activation barrier for diffusion and conductivity. We show that RMD predictions for diffusion and conductivity are comparable to experiments, while results on model systems are consistent with and validated by short (10–100 ps) ab initio molecular dynamics (AIMD). This new ReaxFF for Li_2_S systems enables practical RMD on spatial scales of 10–100 nm (10,000 to 10 million atoms) for the time scales of 20 ns required to investigate predictively the interfaces between electrodes and electrolytes, electrodes and coatings, and coatings and electrolytes during the charging and discharging processes.

## Introduction

Lithium-ion batteries dominate the market for energy storage devices, but the current generation is reaching its maximum possible performance.^1,2^ The increasing demand for portable devices and electric vehicles (EVs)^3,4^ needs the much higher performance of Li–metal anode batteries: high capacity (∼3800–3900 mA h g^–1^), low electrochemical potential (about 3.0 V vs the standard hydrogen electrode, SHE), and low gravimetric density (close to 0.5 g cm^–3^).^5,6^

These promising properties make the application of Li anode batteries indispensable for next-generation energy-storage devices, such as Li–S and Li–air batteries. Despite this promise, Li metal anode batteries have been marginally studied until recently because of safety concerns originating from the high reactivity of Li that causes spontaneous reactions with electrolytes.

In this view, all solid-state batteries (ASSBs), which combine a solid electrolyte with a Li–metal anode, are most promising because they enable achievement of energy storage with higher energy densities and improved safety compared to standard Li-ion cells based on liquid electrolytes. This requires new electrolytes that are stable against a Li–metal anode or, alternatively, a protective layer to stabilize the solid-electrolyte interphase (SEI).

In the field of ionically conductive solids, significant progress has been made recently with the discovery of numerous sulfide-based compounds with superionic conductivities.^7−11^ Argyrodites Li_6_PS_4_XS with X = (Cl, Br, and I) belong to this family of compounds.^12−14^ They are PS_4_-based crystalline Li-rich solids with unusually high Li^+^ mobility. Unfortunately, these electrolytes can react with Li metal anodes. Therefore, we investigate coatings designed to suppress the degradation of the electrolyte while maintaining high Li^+^ conductivity. This provides insight into the stabilization of solid electrolytes at the interface with the Li metal electrodes.

One possible coating at the Li anode is “sulfide-based”. Indeed, ionic materials such as Li_2_S can form during charge/discharge processes during battery operation in sulfur–graphite batteries.^15^ In addition, in a recent paper, we showed the presence of Li_2_S at the termination of {111} and {001} stable surfaces of the Cl–Argyrodite,^16^ which supports the hypothesis of an incipient phase separation working naturally as a possible coating agent. Therefore, Li_2_S represents an interesting starting material to develop and test a comprehensive simulation methodology aimed at understanding how diffusion phenomena occurring at electrolyte/electrode interfaces affect battery performance.

Given the great difficulties for the experimental assessment of the atomistic details of the SEI, we need to use theory to provide a full characterization of the SEI.

Quantum mechanics (QM) on model systems can provide a fundamental atomistic-level description of some of the reactive processes at the interface between Li–metal and the electrolyte, which may help improve our comprehension of solid electrolytes via accurate estimates of ion migration energy barriers, relevant thermodynamic properties, and preferred diffusion pathways.^17−19^ However, the practical size and time scale for QM-based MD (AIMD) are 200–300 atoms (2–3 nm) and 50–100 ps. Instead, we need a tool that allows a reliable prediction of complex reactions involving “sulfides” once the electrode is put in contact with a solid electrolyte. To describe the reactions forming the SEI and its effect on transport during charging and discharging, realistic simulations of the Li anode electrolyte interface require spatial scales of 10–100 nm (10,000 to 10 million atoms) at temperatures of 300 to 400 K for times of 1–20 ns. Consequently, we propose to use the reactive force field (ReaxFF) methodology, which has been employed widely to predict the dynamics of complex multiphase chemical reactions on a large number of systems (>5320 citations), including molecular dynamics (MD) simulations on electrolyte molecules^20,21^ and batteries.^22,23^ ReaxFF uses general bond order-bond distance and bond energy-bond order relationships to describe bond breaking and formation processes,^22^ while optimizing the charge distribution using the charge equilibration (QEq) formalism that uses Gaussian-shaped charges instead of point charges to describe shielding at short distances.^24^ Unfortunately, no ReaxFF is available to describe reactive processes for Li/Li_2_S (or to simulate “sulfide-based” electrolytes such as Argyrodites) systems. Thus, we report here the development of ReaxFF parameters based on comparison to QM using model systems; we apply it to computations on Li_2_S systems with up to eight hundred atoms to provide insight into the properties of the Li_2_S protective layer. We report the formation energies of possible defects in Li_2_S and conduct an exhaustive MD study of anion and cation diffusion in these systems for up to 20 ns over a temperature range of 300 to 900 K. Later, we will use this ReaxFF to obtain a deeper understanding of the complex SEI that should be useful in developing a new generation of highly efficient batteries.

## Computational Methods

### ReaxFF Reactive Force Field Method

The force field optimization procedure we adopted is the one identified by Van Duin for various systems.^22,25^ To optimize the parameters of a force field, it requires a training set of quantum mechanics data for model systems. This training set must contain the kind of data that the force field must be able to produce. Since the major reasons for developing the ReaxFF force field are to predict the mechanisms of Li^+^ diffusion in Li_2_S, the reproduction of the energy cost for Li^+^ vacancies in Li_2_S bulk, and the energy cost for sulfur vacancies and a trivacancy (single unit formula of Li_2_S) by our ReaxFF is of prime concern. The force field must also be able to reproduce: the experimental heat of formation, the expansion/contraction behavior (“roughly” the equation of state (EOS)) of the bulk system, and the interaction between the ionic species Li^+^ and LiS^–^ as a function of distance **r**. The values of energy are considered for a volume variation of Δ*V* ± 10% with respect to zero temperature and zero pressure equilibrium volume *V*_0_.

To build our training set, we considered the energy associated with the processes of formation of vacancies described in the following equations
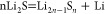
1 =  + *E*^Li^ – *n* is the energy cost for removing the Li atom in Li_2_S corrected for basis set superposition energy (BSSE)^26^ according to the counterpoise method
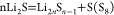
2where corresponding formation energy  =  +  – *n* is the energy cost for the S atom in its bulk with orthorhombic *Pmn*2_1_ symmetry, which contains S_8_ cycles.
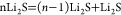
3where  =  +  is the energy cost for remotion of one unit formula of Li_2_S from bulk Li_2_S corrected for BSSE according to the counterpoise method (see Table 1).
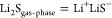
4where  =  –  is the dissociation energy of Li_2_S_gas-phase_ to interacting ions Li^+^ and LiS^–^ at a distance of 4 Å.

**Table 1:** Predicted Formation Energies (in eV) for Vacancies in Li_2_S at the DFT and ReaxFF Levels for the Reactions Adopted in the Training Set

vacancy	PBE0_BS1_	PBE0_BS2_	M06-2XBS2	ReaxFF
Li	5.971	6.030	6.336	5.943
S(S_8_)	5.389	5.452	5.992	5.341
Li_2_S trivac.	7.315	7.516	8.198	6.793
Li^+^–LiS^–^	3.067	3.033	3.134	2.901

The development of this tailored ReaxFF was performed by using the LAMMPS environment. Together with the introduction and optimization of bond, angle, torsion, off-diagonal terms, overcoordination, and under-coordination terms, we also include terms pertaining to the ATOM part of the force field and concerning the Coulomb part described by the QEq approach, for which electronegativity and hardness (χ and *J*) have been optimized^24^ to match well the DFT data adopted as the training set. About the cost function used, the error in the individual quantities is defined as
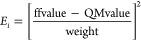
5with **ffvalue**, the ReaxFF energy, and **QMvalue**, the DFT energy as obtained at the UPBE0/Ahlrichs TZVP level, as described in the Computational Methods Section, while the total error or cost function is defined as
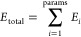
6

The **weight** is 1.0 for each property adopted in the training set, apart for the heat of formation, where the weight we attributed is 3.0, and the energy differences with reference to minimum energy volume *V*_0_ at 0 K in the two cases of 0.99 *V*_0_ and 1.01 *V*_0_, where a weight of 0.2 was assigned. A code by van Duin implementing the methodology reported in refs (22) and (25) was adopted, which was previously used in a number of published papers. The electronegativity and hardness were optimized to reproduce the interaction between free ions Li^+^ and LiS^–^. Moreover, the charges on atoms closer to Li vacancies have been optimized to reproduce the Mulliken charges obtained in DFT calculations at the PBE0/Ahlrichs TZVP level.

### Quantum Mechanical Calculations

Unrestricted PBE0 (as implemented in the Crystal periodic program^27,28^) calculations were performed together with the Lichanot basis set^29^ and the Ahlrichs split valence triple-ζ basis sets plus polarization (TZVP) for all the elements.^30^ The M06-2X Minnesota high-nonlocality functional with doubled nonlocal exchange, 2X, was also adopted to account for noncovalent interactions^31^ The truncation criteria of the Coulomb and exchange for infinite lattice series were controlled by five thresholds: *T*_*i*_ = 8 (for *T*_1_–*T*_4_) and *T*_5_ = 16. The convergence threshold on energy for the self-consistent-field (SCF) procedure was set to 1 × 10^–8^ hartree for structural optimizations. A pruned Becke grid with 75 radial and 974 angular points in regions relevant for chemical bonding was used. Reciprocal space sampling is based on a regular Pack–Monkhorst^32^ grid centered at the Γ point, with a shrinking factor of 6 along each vector. For DFT calculation of defects in bulk Li_2_S, we adopted a 2 × 2 × 2 supercell (96 atoms) of the conventional cubic bulk cell in the *Fm*3̅*m* symmetry group. To estimate the dissociation energy of Li_2_S_gas-phase_ to interacting ions Li^+^ and LiS^–^, we used single point PBE0/Ahlrichs split valence triple-ζ basis sets plus the polarization (TZVP) level on the geometry obtained at the PBE0/QZVP level.

### Molecular Dynamics Simulations

On both perfect bulk and defective model systems, we performed minimization and molecular dynamics simulation by employing our newly optimized ReaxFF, using the LAMMPS code.^33^ Initial structures started with the DFT minimum energy structures for the cubic cell of Li_2_S, as described above for both the Frenkel defects and for vacancies. From these starting points, we built 3 × 3 × 3 supercell structures, including a lithium vacancy for systems containing 324 atoms and 4 × 4 × 4 supercells containing 768 atoms for systems containing Frenkel defects. The diffusion of Li and sulfur atoms was investigated using molecular dynamics. MD simulations were performed with the following steps:

- Minimization using ReaxFF;
- Simulations at constant volume with a thermostat for 10 ps at 10 K to generate initial velocities for atoms;
- Heating the structure from 10 K to *T* = 300 K using NVT for 100 ps;
- Additional MD simulations at 500, 600, 700, and 800 K for 1 ns;
- NVT molecular dynamics simulations for production at 300 K, applying the Nose–Hoover thermostat. The same for all other temperatures 500, 600, 700, and 800 K for 1 ns.

From the Nernst–Einstein diffusion equation, we can obtain the diffusion coefficient (*D*) from the mean square displacement (log MSD) versus time (log *t*) at various temperatures
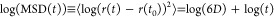
7where MSD indicates the average change for each Δ*R*^2^ for Δ(*t*) averaged over the whole trajectory. In the limit of Fickian-régime, we recover the *D* coefficient from the slope of log MSD vs log *t*.

We also computed the self-diffusion *D* coefficient by applying an electric field **E** to evaluate the mobility μ after attaining a constant velocity *v* = μ*E*. We then obtained the diffusion coefficient *D* from the eq 8
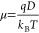
8where *q* is the charge averaged over all Li atoms during the 1 ns trajectories, and *k*_B_ is the Boltzmann constant. Then, we compute the ion conductivity (σ) from eq 9
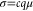
9where *c* is the concentration of vacancies (cm^–3^ of Li-ion), and *q* (Coulomb) is the charge for Li averaged over all Li-ion in the system and between the first and last frame of trajectory.^34,35^

The activation energy *E*_a_ was calculated from the Arrhenius equation for conductivity
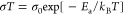
10

We applied an electric field **E** along the *x*, *y*, and *z* directions with various fields. We report the averaged diffusion coefficient. This method for determining *E*_a_ and the autodiffusion coefficient will be indicated hereafter as Method 1 or M1.

The activation energy *E*_a_ for the Li diffusion in both vacancy and interstitial mechanisms was also evaluated by structural analysis (Method 2, M2 hereafter) by considering the displacement of Li ions and the consequent change of coordination at sulfur atoms. The number of diffusion events in units of time (s^–1^) provides the velocity of migration at different temperatures along the molecular dynamics trajectory. The velocity is a function of temperature according to the following Arrhenius equation
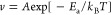
11where the ratio between the number of events of vacancy migration in seconds is on the log scale versus the inverse of temperature. The slope of the log plot gives the *E*_a_/*k*_B_ factor for Li diffusion.

## Results and Discussion

### Training Set: DFT and ReaxFF Predictions

The total energy for vacancy formation defined in eqs 1–3 for various levels of DFT and our ReaxFF is reported in Table 1. The various functionals lead to similar results, and the Lichanot basis set appears large enough to provide reliable thermodynamical results. For the Li vacancy case, we use an atomic reference state (according to eq 1), while for S, we use the crystal in the orthorhombic *Pmn*2_1_ space group (according to eq 2). We see that the predictions from our customized ReaxFF are in good agreement with QM. Similarly, the prediction for an ionic behavior between the ionic species Li^+^ and LiS^–^ with distance **r** matches well the DFT data shown in Table 1 for a system with ions interacting at a distance of 4.0 Å.

We underline the high accuracy of the present ReaxFF parametrization: the error in the PBE0_BS2_ DFT database is below 0.1 eV for the formation energy of both Li and S vacancies and for the dissociation of the Li_2_S molecule in the gas phase, achieving chemical accuracy for quantities on the order of several eV. Only the formation energy of the trivacancy is somewhat underestimated. The optimized ReaxFF was based on systems with various types of defects and was adopted for NVT molecular dynamics on systems with different types and concentrations of defects at a range of temperatures.

We also built models with cation Frenkel defects where an intersticial Li^+^ ion is present together with a “far away” vacancy to guarantee the neutrality of the systems (Frenkel *V*_Li_^–^ Li^+^ pairs). In addition, we considered neutral vacancies in bulk Li_2_S.

To investigate vacancy diffusion, we considered three vacancy densities 1%, 2%, and 3% to determine *E*_a_ for Li^+^ ion diffusion by structural analysis according to method M1. To obtain results that are independent from a specific configuration of defects, the number of events per time unit has been averaged over three different configurations of defects to obtain the *E*_a_ value. The data come from 1 ns molecular dynamics run at 300, 400, 500, 600, 700, and 800 K. Unfortunately, the number of events at the lowest concentration of defects (1%) is so low that the predicted conductivity is less reliable. Instead, we use *E*_a_ from the higher concentrations to estimate the 300 K conductivity.

In no case are diffusion phenomena observed at 300 K; the first events are visible in NVT molecular dynamics performed at 600 K. This already provides an indication of a quite high barrier to Li diffusion. The diffusion of Li through the vacancy mechanism follows the path −8c–8c–8c– sites. The structure of a 2 × 2 × 2 supercell (96 atoms) of the conventional cubic bulk cell in the *Fm*3̅*m* symmetry group is reported in Figure 1 of Supporting Information, together with the identification of sites in Figure 2 of the same file.

**Figure 1:**
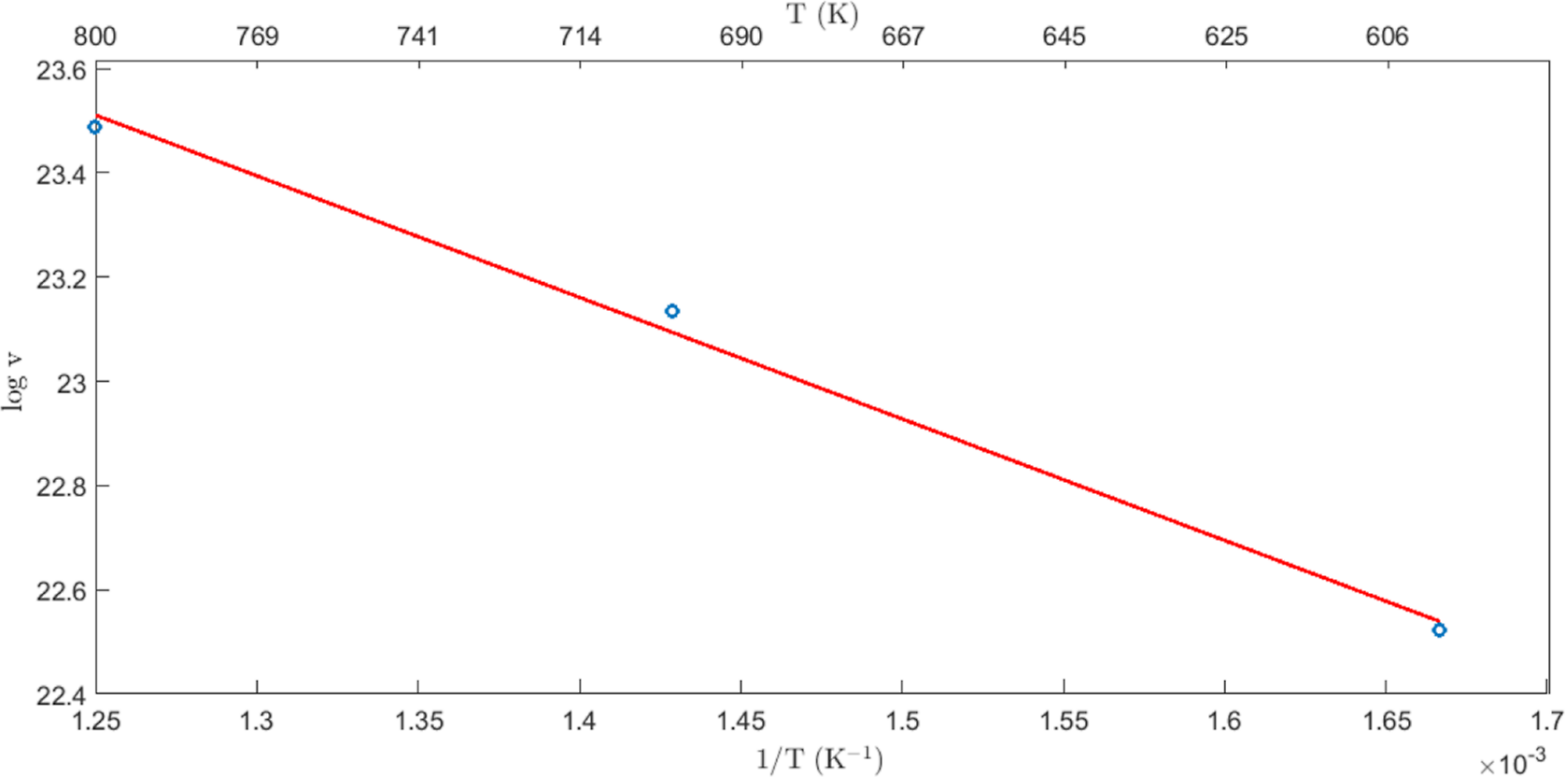
Log plots of Li-ion diffusion vs 1/*T* (K) for Li_2_S systems containing 3% of Li vacancies; the open circle represents the outcome of simulations; the straight line is the interpolating first order polynomial.

The log plots of Li-ion diffusion vs 1/*T* (K) in the case of Li_2_S systems containing 3% of Li vacancies are reported in Figure 1; the open circle represents the outcome of simulations, and the straight line is the interpolating first-order polynomial. The predicted energy barrier for the hopping of a Li to a vacant Li site is *E*_a_^vac^ = 0.201 eV. This prediction can be compared to the DFT counterpart,^36^ as discussed below.

We also performed MD simulations at 600, 700, and 800 K in selected cases for 20 ns. The outcomes for *E*_a_ are in line with previous outcomes from 1 ns NVT simulations.

We built a system containing 786 atoms with 8 Frenkel defects (i.e., Li ions in interstitial positions are associated with an equal number of vacancies). In this case, the concentration of interstitial defects Li^+^ ∼ 1.01% is the same density of holes, representing its negative defect. NVT dynamics were carried out for 1 ns in the same temperature range of 300–800 K adopted for vacancy defects. The analysis of trajectories allowed us to identify the diffusion mechanism via the so-called knock-on mechanism, where an interstitial Li ion (in the octahedral site) kicks a Li sitting on a regular lattice site to an interstitial sit and so until the vacancy is annihilated; the diffusion of Li in our trajectories occurs along the path −8c–4b–8c– sites. Similarly to what we did for the vacancy diffusion mechanism, we obtained the *E*_a_^interstitial^ value for the diffusion process from the Arrhenius plot by running 1 ns molecular dynamics simulations at 300, 400, 500, 600, 700, and 800 K. The plot of interest is reported in Figure 3. We obtained that *E*_a_^interstitial^ is equal to 0.459 eV.

**Figure 2:**
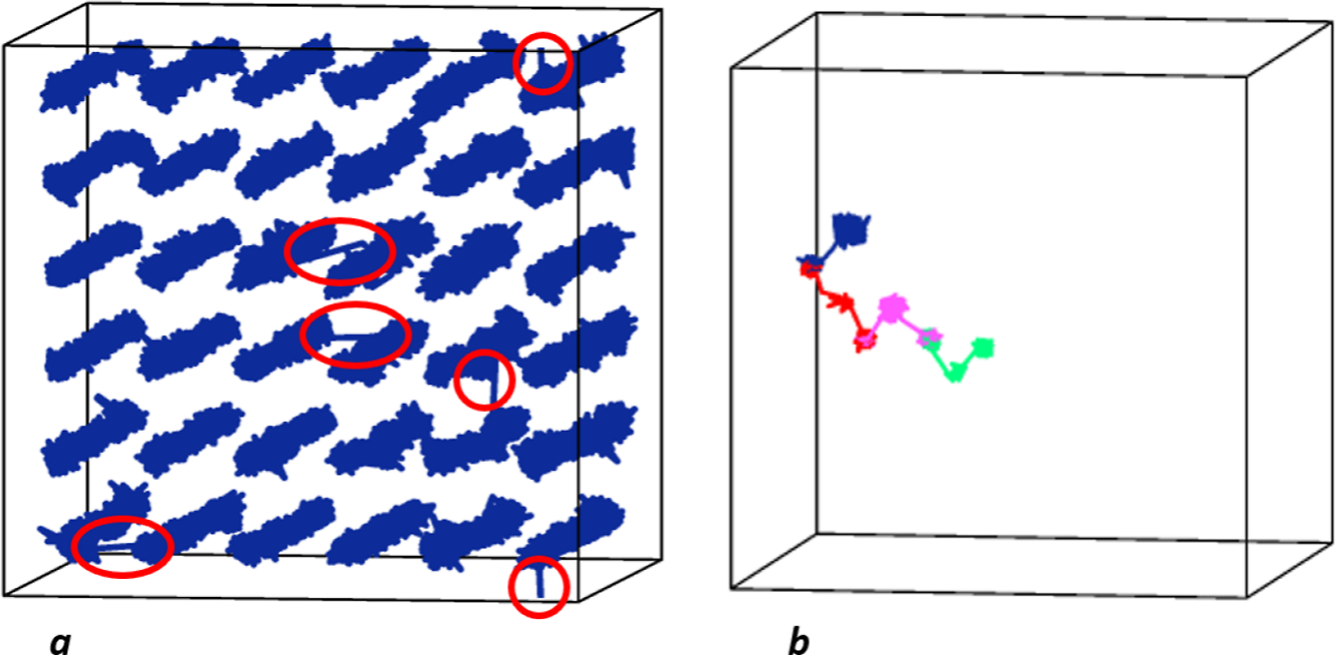
(a) Li-ion trajectories for Li_2_S with 3% of Li vacancies at 600 K for 1 ns RMD; the Li-ion jumps to a hole site are highlighted by red circles; (b) diffusion path for the knock-on mechanism identified in Li_2_S with Frenkel defects (2% of Frenkel pairs) at 600 K for 1 ns run.

**Figure 3:**
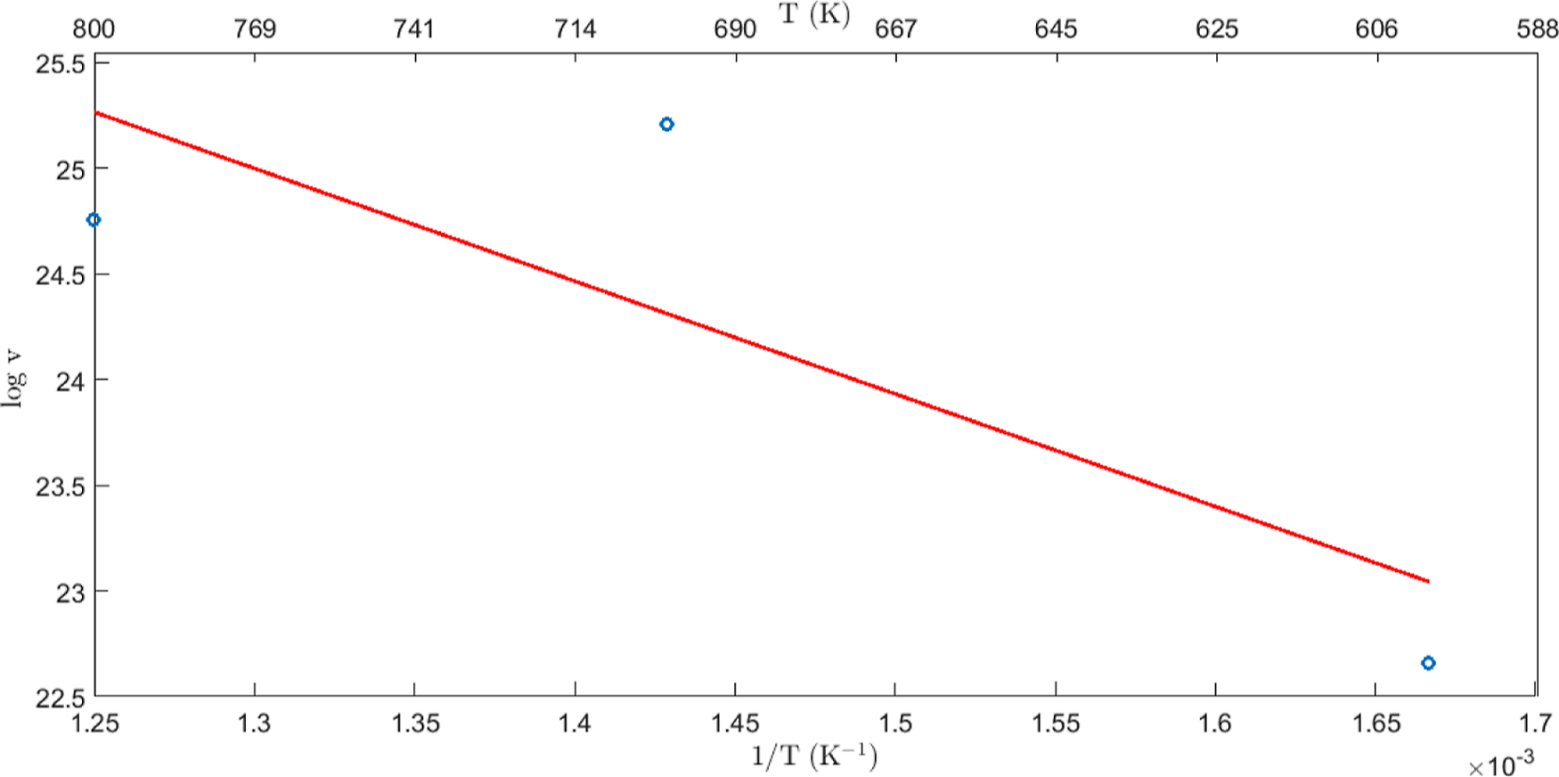
Log plots of Li-ion diffusion vs 1/*T* (K) for the knock-on mechanism in the case of Li_2_S systems containing ∼1.01% of Li interstitial defects. The open circles represent the outcome of simulations; the straight line is the interpolating first-order polynomial.

Therefore, we can use the conventional cation vacancy model to interpret the conductivity of lithium sulfide and the collinear type of interstitial mechanism. Li-ion diffusion trajectories over a 1 ns simulation are shown in Figure 2a,b, respectively, for each mechanism.

Comparing the diffusion barriers for both mechanisms, we infer that ionic conductivity in Li_2_S may occur via the migration of both interstitial Frenkel Li^+^ pairs and vacancies, but the migration through vacancies is favored.

### The Effect of Defect Density on Diffusion Coefficients and Activation Energy

AIMD simulations for 40 ps were reported previously for Li_2_S with a single Li vacancy out of 32 unit formulas at temperatures from *T* = 300 to 1300 K. Unfortunately, the MD needs to be > 1 ns to obtain reliable diffusion coefficients. In contrast, using ReaxFF, we were able to do MD up to 1 ns to determine the effect of defect density on the self-diffusion coefficient (*D*). This leads to the activation barrier *E*_a_, mobility μ, and conductivity σ for vacancy concentrations of 0.3%, 2%, and 3%. The 0.3% model, which contained one vacancy for every 324 atoms, will be adopted as the case for very low defect density. Under the application of a static force *F* = *q* × *E* to each particle, we determined the displacement of each Li ion along the 1 ns trajectory for all defect-containing systems. The velocity was determined as an average over the displacements along all three Cartesian directions for the cubic symmetry Li_2_S. Reactive molecular dynamics (RMD) were carried out for 1 ns at various temperatures in the range 600–900 K. Depending on the concentration of defects in the system, we obtained a constant velocity of particles at various values of the applied electric field. Field strengths of 0.01 and a “weak” 0.0005 V/Å were applied to 2% and 3% vacancies in Li_2_S to obtain a constant velocity of Li^+^ ion migration. Stronger fields, of strength 0.03 V/Å, were applied in the case of 0.3% Li vacancy concentration to observe diffusion phenomena at the nanosecond time scale.

**Figure 4:**
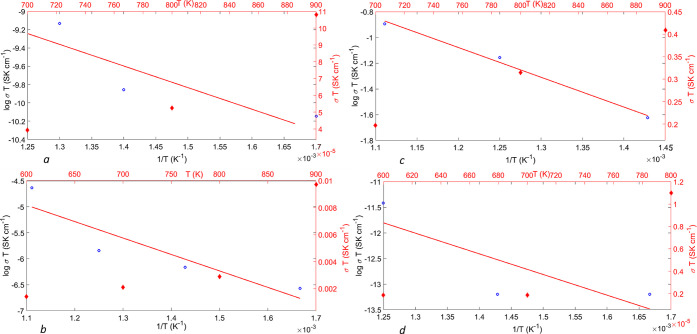
(a) Log plot of conductivity of Li-ion (the right axis) vs 1/*T* (K) (bottom axis) and conductivity (σ*T* left axis) vs *T* (top axis) for a model of Li_2_S containing a vacancy at a very low dilution of ∼0.3%. The applied field is 0.03 V/Å. The open circles represent the (log σ*T* points); the straight line is the interpolating first-order polynomial, whereas the diamonds represent the conductivity (σ*T*) outcomes of simulations. (b) Log plot of conductivity of Li-ion (the right axis) vs 1/*T* (K) (bottom axis) and conductivity (σ*T* left axis) vs *T* (top axis) for a model of Li_2_S containing 2% vacancy. The applied electric field is 0.01 V/Å. (c) Log plot of conductivity of Li-ion (the right axis) vs 1/*T* (K) (bottom axis) and conductivity (σ*T* left axis) vs *T* (top axis) for a model of Li_2_S containing vacancies with a density of 3%. The applied field is 0.0005 V/Å. (d) Log plot of conductivity of Li-ion (the right axis) vs 1/*T* (K) (bottom axis) and conductivity (σ*T* left axis) vs *T* (top axis) for a model of Li_2_S with a density of 1% interstitial Li^+^ ion or 2% of Frenkel pairs, as described in the text. The applied electric field is 0.01 V/Å.

The plots of log σ*T* vs 1/*T* and the plot of σ*T* vs *T* are reported in Figure 4a–c for the Li vacancy cases; while Figure 4d reports the conductivity for interstitial defects.

The energy barrier *E*_a_^vac^ for each vacancy density was derived from eq 10 leading to *E*_a_^vac^ = 0.268 eV for 0.3% defect density and *E*_a_^vac^ = 0.270 eV for 2% vacant Li sites. The result at low vacancy density indicates that the electric field has little effect on the barrier. For a higher vacancy density (3%), our Method 1 predicts a lower activation barrier of *E*_a_^vac^ = 0.200 eV, which interestingly points to cooperative phenomena from vacancy–vacancy interactions. These results agree with the previous predictions of *E*_a_^vac^ = 0.201 eV from the structural analysis of vacancy-rich Li_2_S models (crystals with 3% defects).

The predicted Li ion diffusivity and conductivity determined according to eqs 8 and 9 are reported in Table 2 at 700 K for each defective system, together with the activation energies for diffusion according to eq 10; the value for σ*T* extrapolated to 300 K is 9.652 × 10^–8^ S K cm^–1^ for 0.3% defect density, as reported in the last column of Table 2, together with the values for various defect densities.

**Table 2:** Li-Ion Diffusivity (*D*) and Conductivity (σ) Per Formula Predicted From Our MD Simulations for 1 ns at 700 K and Extrapolated to 300 K^a^

Li_2_S	*D*	σ	*E*_a(M1)_	*E*_a(M2)_	σ*T*_300 K_
0.3% Li_vac_	3.40 × 10^–8^	5.614 × 10^–8^	0.268		9.652 × 10^–8^
2% Li_vac_	8.83 × 10^–7^	3.008 × 10^–6^	0.270		5.927 × 10^–6^
3% Li_vac_	5.43 × 10^–5^	2.814 × 10^–4^	0.199	0.201	2.400 × 10^–3^
1% interst.	3.49 × 10^–9^	2.651 × 10^–9^	0.348	0.459	1.659 × 10^–9^
2% Frenkel pair	6.45 × 10^–6^	9.924 × 10^–6^	0.215		2.628 × 10^–5^

a *D* is reported in (cm^2^ s^–1^), σ in (S cm^–1^), and *E*_a_ in eV.

For the interstitial mechanism (Frenkel V_Li_^–^ Li^+^ pairs), the diffusion coefficient *D* = 3.49 × 10^–9^ cm^2^ s^–1^ at 700 K from application of an electric field of 0.01 V/Å, as reported in Table 2, with 1% Li^+^ and *D* = 6.45 × 10^–6^ (cm^2^ s^–1^) with 2% total density of defects, where we also consider the vacancies Li_vac_. when the Frenkel pair is considered. This is consistent with the higher activation barrier we predict for the interstitial mechanism compared to the vacancy-hopping mechanism. For the Frenkel pair defects, both vacancy diffusion and interstitial migration may occur, but the vacancy diffusion will be faster since the diffusion of Frenkel defects happens via holes.

Considering only the vacancy mechanism for 2% Li_vac_, we predict *D* = 8.83 × 10^–7^ cm^2^ s^–1^ using method M1. In the case of Frenkel V_Li_^–^ Li^+^ pairs, the contribution from hopping, *D* = 6.45 × 10^–6^ (cm^2^ s^–1^), is larger than the *D* = 8.83 × 10^–7^ cm^2^ s^–1^) vacancy hopping with the same 2% Li_vac_ density of defects. This can be justified by the interaction of hole defects and interstitial defects that favor increased mobility. The NEB and AIMD lead to a diffusion activation barrier of *E*_a_^vac^ = 0.2 eV for a single vacancy, which is in very good agreement with our ReaxFF predictions despite the very short AIMD.^36,37^ The paper titled “Theoretical study of superionic phase transition in Li_2_S″ published in Scientific Reports^37^ also reports 50 ps AIMD at temperatures T = 300, 600, 750, 830, 900, 1050, 1170, and 1300 K on the Li_2_S system in a small 2 × 2 × 2 supercell. Even if the latter paper aims at modeling a superionic phase transition above 900 K, the authors declared that “only a few number of Li vacancy hoppings were observed for *T* = 300, 600, and 750 K”; moreover, it is found that Li transport occurs mainly via Li vacancy hopping between regular Li sites (so-called 8c sites) at low temperatures such as *T* = 830 K. At higher temperatures, anharmonic elongation in Li ion positions appears. Although Li transport still takes place mainly via Li vacancy hopping between 8c sites, there are few Li jumps between 8c and interstitial defective sites (so-called 4b sites) at *T* = 900 K”. The paper clearly identifies the two mechanisms we report in our manuscript: hole-hopping (vacancy diffusion) at lower temperatures and interstitial diffusion (“Li jumps between 8c and interstitial defective sites 4b”), and the latter diffusion became obviously visible at higher temperatures. From the Arrhenius plot of the diffusion coefficient reported in the paper of Kaghazchi et al. (ln *D* vs 1/*k*_B_*T*), we recovered at 830 K (the lowest temperature datum the authors reported) *D* ∼ 3.2076 × 10^–6^ cm^2^/s, which can be compared with our value of 1.2316 × 10^–6^ cm^2^/s at 800 K for 2% vacancy density. If we consider that the concentration of vacancies in that paper “vac & dis” as charge carriers are both Li vacancy and disorder (interstitial Li) is about 2% (2/96, 96 is the number of atoms in a supercell 2 × 2 × 2) and that at “low temperature” the largest part of diffusion occurs via vacancy as the authors claim, we can assume that our results are in the same ballpark as AIMD. The tiny discrepancies between AIMD and RMD can be interpreted in terms of both the dimensions of the adopted models and the employment of the GGA (PBE) functional in the case of AIMD toward our ReaxFF, which we parametrized against a DFT approach using a hybrid exchange–correlation functional, which is recognized to be much more accurate than any gradient-corrected xc-functional.

We predict a strong dependence of diffusion coefficients upon the vacancy density, with *E*_a_^vac^ decreasing dramatically from 2% to 3% up to *E*_a_^vac^ = 0.2 eV in the latter case, corresponding to an intrinsic conductivity σ*T* = 10^–3^ S K cm^–1^ at 300 K.

Experimental values for ionic conductivity σ*T* (at 298 K) of Li_2_S^38,39^ are spread over a huge range from σ*T* = 10^–5^ to 10^–10^ (S K cm^–1^) depending on sample preparation. The Li^+^ conductivity in films depends on the sputtering technique, the temperature of annealing, grain size, and the deposition parameters that affect the morphology of the sample.^40^ The change in the diffusion activation energy upon annealing can be attributed to a change in mechanism from dislocation-driven to grain-boundary-driven conduction and/or a space charge effect with a changing segregation energy. Experiments identify a range of 10^–10^–3 × 10^–8^ S cm^–1^ for conductivity in film of Li_2_S at 298 K.^41^ We predict the self-diffusion coefficient reported in Table 2 at 700 K for various defect concentrations, including vacancies and Frenkel pair defects. The same has been done for temperatures in the range 600–900 K. The values have also been extrapolated at 300 K to compare the simulation results to values obtained in battery operation conditions. Since the experimental conductivity is the product of intrinsic diffusion coefficients times the number of carriers, thus assuming grain boundaries as the most common defects, we estimated the grain size, concluding that the samples in refs (38) and (39) had a grain size of the order of tens of nm, whereas the samples in ref (41) had a grain size of the order of hundreds of microns. This leads to an estimate of semiquantitative agreement between our predictions and the experiment.

Up to this point, we considered only the barrier for defect migration, but before comparing the predicted conductivity to the experiment, we recall that high conductivity can arise from both lower defect formation energies and lower cation migration energies. Of course, to estimate the total activation energy of diffusion for the vacancy and interstitial Li^+^ mechanisms, we must consider both the formation and migration processes of defects. Based on ReaxFF, the formation energy is 2.33 eV for the Frenkel defect in Li_2_S bulk  compared with  = 5.943 eV for Li vacancy formation (reported in Table 1). The total activation energy of diffusion for vacancy and interstitial Li^+^ diffusion mechanisms is predicted to be *E*_all_^vac^ =  + *E*_a_^vac^ ∼ 6.144 eV, which can be compared with *E*_all_^intersticial^ = *E*_a_^intersticial^ + 1/2 ∼ 1.624 eV. Therefore, even if the barrier for self-diffusion is lower for an isolated vacancy, the global cost of the migration process in Li_2_S bulk via the two investigated mechanisms seems to be favorable for Frenkel interstitial migration. But the conductivity process may be dominated by grain boundaries, where the largest fraction of defects reside. In that case, the formation energy of defects and vacancies is surely lower. In the research on superionic conductors, the challenge to attaining a very high intrinsic conductivity in the material is identifying new materials where the formation energy of defects is also reasonably in bulk.^42^ We also analyzed the MSD of Li^+^ to determine the self-diffusion coefficients according to eq 7, with results confirming the values determined from the application of the static electric field, *F* = *q* × *E*. The analysis of MSD for sulfur ions reveals that no significant anion diffusion is observed at 300 K and higher temperatures or as a function of the electric field strength.

## Conclusions

In this work, we optimized ReaxFF to simulate both pristine bulk and defective Li_2_S systems containing both Li-ion vacancies and Frenkel pair defects. The new ReaxFF was validated against QM on model systems. It reproduces well

- the equation of state for Li_2_S,
- the cost for formation of Li vacancy, sulfur vacancy, and Li_2_S trivacancy.

We considered two Li^+^ diffusion mechanisms: vacancy and interstitial at various defect densities. We predicted the self-diffusion coefficient and conductivity with a reactive molecular dynamics simulation of up to 20 ns, both with and without applied electric field gradients. We predict the activation energy for Li-ion migration via the vacancy mechanism to be *E*_a_^vac^ = 0.20–0.27 eV. For interstitial Li-ion migration, we find a larger *E*_a_^interstitial^ = 0.35–0.45 eV using two methodologies: structural analysis and applied electric field (along the three orthogonal directions).

In the presence of a Frenkel pair, we found that defect diffusion occurs via holes. The good agreement with 50 ps DFT-based AIMD confirms the accuracy of our new ReaxFF. However, the relative order of the formation energy of the defects in the bulk Li_2_S is reversed. Therefore, to estimate the total activation energy of bulk diffusion for both vacancy and interstitial Li^+^ mechanisms, we included both the processes of defect formation and migration to obtain *E*_a_^vac^ =  + *E*^vac^ ∼ 6.144 eV, which can be compared with *E*_a_^intersticial^ = *E*^intersticial^ + 1/2 ∼ 1.624 eV. However, the conductivity process in experimental films is likely dominated by grain-boundary defects. For grain boundaries, the formation energy for defects and vacancies is lower, leading to higher conductivity for defective materials.

We predict the diffusion coefficient *D* and conductivity σ in the range of 600–900 K from 1 to 2 ns molecular dynamics and extrapolated them to *T*_300 K_ for systems with various defect densities. We predict a strong dependence of diffusion coefficients on the vacancy density. *E*_a_^vac^ decreasing dramatically for very defective systems. Indeed, we show that the intrinsic conductivity of a defective system can reach values as low as *E*_a_^vac^ = 0.2 eV, corresponding to intrinsic conductivity as high as σ*T* = 10^–3^ S K cm^–1^. These predictions constitute relevant groundwork toward a better understanding of ionic transport in Li-ion conductors at the electrolyte/anode and electrolyte/cathode interfaces. In the present paper, we identify a useful approach that allows the community to follow a reaction in a battery system over a time scale of nanoseconds. This, we believe, is the more valuable as we parametrized our ReaxFF against a DFT approach using a hybrid exchange-correlation functional, which is recognized to be much more accurate than any gradient-corrected xc-functional. In doing so, we thus go beyond the usual DFT static or dynamic simulations reported in the literature. In this research on superionic conductors, our calculations identify as the main challenge the identification of new materials, where the defect formation energy is reasonably low while leading to a very high intrinsic conductivity in the bulk materials.

## Supporting Information Available

ReaxFF force field optimized for Li/Li_2_S systems and adopted in all calculations reported in the present manuscript; 2 × 2 × 2 supercell (96 atoms) of the conventional cubic bulk cell in the *Fm*3̅*m* symmetry group; and sites 4b and 8c in 2 × 2 × 2 supercell of the conventional cubic bulk cell in the *Fm*3̅*m* symmetry group (PDF)
Folder containing input files (ZIP)
