# Supplementary material for: Understanding Ionic Diffusion Mechanisms in Li2S Coatings for Solid-State Batteries: Development of a Tailored Reactive Force Field for Multiscale Simulations
Source: J Phys Chem C Nanomater Interfaces. 2023 Nov 15;127(47):22880–8. doi: 10.1021/acs.jpcc.3c04991 (PMC10694816; doi:10.1021/acs.jpcc.3c04991)
Supplement: Supplementary file 1 — jp3c04991_si_001.pdf [file jp3c04991_si_001.pdf]

Understanding Ionic Diffusion Mechanisms in Li_2S Coatings for Solid-state Batteries: Development of a Tailored Reactive Force Field for Multiscale Simulations

Maddalena D'Amore,[*,†] Moon Young Yang,[‡] Tridip Das,[‡] Anna Maria Ferrari,[†] Kim Minho,[‡] Riccardo Rocca,[†,¶] Mauro Sgroi,[†] Alessandro Fortunelli,[§] and William A. Goddard III[‡]

†*Dipartimento di Chimica, Università di Torino, Via P. Giuria 5, 10125 Torino, Italy*

‡*Materials and Process Simulation Center (139-74), California Institute of Technology, Pasadena, California 91125*

¶*Centro Ricerche FIAT S.C.p.A., Strada Torino 50, 10043, Orbassano (To), Italy*

§*CNR-ICCOM, Consiglio Nazionale delle Ricerche, via Giuseppe Moruzzi 1, 56124, Pisa, Italy*

E-mail: maddalena.damore@unito.it

In the following, we report in LAMMPS code format, the optimized parameters for new reactive force field (ReaxFF) which we obtained for pristine and defective Li_2S. This reactive force field was adopted in all minimizations and Molecular Dynamics simulations reported in the manuscript. We also report the structure of $2 \times 2 \times 2$ supercell (96 atoms) of Li_2S conventional cubic bulk cell in the $Fm\bar{3}m$ symmetry group and identified the sites 4b and 8c involved in Li-ion diffusion mechanisms we investigated.

Supporting Information Available

- Reaxff_atomref_MD.fort4 ;

Reactive MD-force field: Li2S pristine and defected, Li, S, Li2S vacancies

39 ! Number of general parameters

50.0000 !p(boc1)

9.5469 !p(boc2)

26.5405 !p(coa2)

1.5105 !p(trip4)

6.6630 !p(trip3)

70.0000 !kc2

1.0588 !p(ovun6)

4.6000 !p(trip2)

12.1176 !p(ovun7)

13.3056 !p(ovun8)

-10.1292 !p(trip1)

0.0000 !Lower Taper-radius (swa)

10.0000 !Upper Taper-radius (swb)

0.0000 !n. u.

33.8667 !p(val7)

6.0891 !p(lp1)

1.0563 !p(val9)

2.0384 !p(val10)

6.1431 !n. u.

6.9290 !p(pen2)

0.3989 !p(pen3)

3.9954 !p(pen4)

0.0000 !n. u.

5.7796 !p(tor2)

10.0000 !p(tor3)

1.9487 !p(tor4)

0.0000 !n. u.

2.1645 !p(cot2)

1.5591 !p(vdW1)

0.1000 !Cutoff for bond order*100 (cutoff)

2.1365 !p(coa4)

0.6991 !p(ovun4)

50.0000 !p(ovun3)

1.8512 !p(val8)

0.0000 !n. u.

0.0000 !n. u.

0.0000 !n. u.

0.0000 !n. u.

2.6962 !p(coa3)

11 ! Nr of atoms; atomID;ro(sigma); Val;atom mass;Rvdw;Dij;gamma

alfa;gamma(w);Val(angle);p(ovun5);n.u.;chiEEM;etaEEM;n.u.

ro(pipi);p(lp2);Heat increment;p(boc4);p(boc3);p(boc5),n.u.;n.u.

p(ovun2);p(val3);n.u.;Val(boc);p(val5);n.u.;n.u.;n.u.

C 1.3825 4.0000 12.0000 1.9133 0.1853 0.9000 1.1359 4.0000

9.7602 2.1346 4.0000 33.2433 79.5548 5.8678 7.0000 0.0000

1.2306 0.0000 199.0303 8.6991 34.7289 13.3894 0.8563 0.0000

-2.8983 2.5000 1.0564 4.0000 2.9663 0.0000 0.0000 0.0000

H 0.7853 1.0000 1.0080 1.5904 0.0419 1.0206 -0.1000 1.0000

9.3557 5.0518 1.0000 0.0000 121.1250 5.3200 7.4366 1.0000

-0.1000 0.0000 54.5000 1.9771 3.3517 0.7571 1.0698 0.0000

-15.7683 2.1488 1.0338 1.0000 2.8793 0.0000 0.0000 0.0000

O 1.2477 2.0000 15.9990 1.9236 0.0904 1.0503 1.0863 6.0000

10.2127 7.7719 4.0000 36.9573 116.0768 8.5000 8.9989 2.0000

0.9088 1.0003 60.8726 20.4140 3.3754 0.2702 0.9745 0.0000

-3.6141 2.7025 1.0493 4.0000 2.9225 0.0000 0.0000 0.0000

S 1.9186 2.0000 32.0600 1.6516 0.4937 0.7530 1.6593 6.0000

9.0227 4.9055 4.0000 30.0000 112.1416 6.5645 9.0100 2.0000

1.0000 3.4994 65.0000 12.0000 22.1978 15.3230 0.9745 0.0000

-15.7363 2.8802 1.0338 6.2998 2.8793 0.0000 0.0000 0.0000

Mo 2.4695 5.6375 95.9400 1.8471 0.3413 1.0000 0.1000 6.0000

13.1958 44.8826 4.0000 0.0000 0.0000 0.7695 6.0677 0.0000

0.1000 0.0000 152.6300 3.4529 0.0722 3.1767 0.8563 0.0000

-17.9815 3.1072 1.0338 8.0000 3.4590 0.0000 0.0000 0.0000

Ni 1.8201 2.0000 58.6900 1.9449 0.1880 0.8218 0.1000 2.0000

12.1594 3.8387 2.0000 0.0000 0.0000 4.8038 7.3852 0.0000

-1.0000 0.0000 95.6300 50.6786 0.6762 0.0981 0.8563 0.0000

-3.7733 3.6035 1.0338 8.0000 2.5791 0.0000 0.0000 0.0000

Li 1.9814 1.0000 6.9410 1.8000 0.2939 0.9387 -0.1000 1.0000

9.0616 1.3258 1.0000 0.0000 0.0000 -3.0000 10.0241 0.0000

-1.0000 0.0000 37.5000 5.4409 6.9107 0.1973 0.8563 0.0000

-2.5068 2.2989 1.0338 1.0000 2.8103 1.3000 0.2000 13.0000

B 1.5530 3.0000 10.8110 1.6512 0.1000 0.9480 1.0000 3.0000

10.3025 2.3647 3.0000 0.7036 80.0000 4.0000 7.0000 0.0000

-1.3000 0.0000 151.3700 7.6069 1.9324 1.0943 0.0000 0.0000

-3.1611 4.0000 1.0564 3.0000 2.8413 0.0000 0.0000 0.0000

F 1.1620 1.0000 18.9984 1.5562 0.1213 0.5000 -0.1000 7.0000

10.2712 7.5000 1.0000 9.2533 0.2000 9.0000 8.0000 0.0000

-1.0000 3.4296 18.0000 6.9821 4.1799 1.0561 0.0000 0.0000

-7.3000 2.6656 1.0493 4.0000 2.9225 0.0000 0.0000 0.0000

P 1.5994 3.0000 30.9738 1.7000 0.1743 1.0000 1.3000 5.0000

9.1909 14.9482 5.0000 0.0000 0.0000 1.8000 7.0946 0.0000

-1.0000 25.0000 1.5000 0.2187 21.4305 15.1425 0.0000 0.0000

-3.9294 3.4831 1.0338 5.0000 2.8793 0.0000 0.0000 0.0000

N 1.6157 3.0000 14.0000 1.9376 0.1203 1.0000 1.2558 5.0000

9.4267 26.8500 4.0000 8.6294 100.0000 7.6099 7.7500 2.0000

1.0439 0.1000 119.9837 1.7640 2.7409 2.3814 0.9745 0.0000

-6.5798 4.4843 1.0183 4.0000 2.8793 0.0000 0.0000 0.0000

51 ! Nr of bonds; at1;at2;De(sigma);De(pi);De(pipi);p(be1);p(b

p(be2);p(bo3);p(bo4);n.u.;p(bo1);p(bo2)

1 1 156.6463 99.9144 80.0715 -0.8028 -0.4648 1.0000 37.6741 0.4292

0.4291 -0.1024 9.2608 1.0000 -0.0500 6.8233 1.0000 0.0000

1 2 170.2316 0.0000 0.0000 -0.5931 0.0000 1.0000 6.0000 0.7140

5.2267 1.0000 0.0000 1.0000 -0.0500 6.8315 0.0000 0.0000

2 2 156.0973 0.0000 0.0000 -0.1377 0.0000 1.0000 6.0000 0.8240

2.9907 1.0000 0.0000 1.0000 -0.0593 4.8358 0.0000 0.0000

1 3 146.4444 155.0589 61.1630 -0.9812 -0.0842 1.0000 11.0135 0.1494

2.3426 -0.2536 6.5307 1.0000 -0.1973 6.1418 0.0000 0.0000

3 3 60.1463 176.6202 51.1430 -0.2802 -0.1244 1.0000 29.6439 0.9114

0.2441 -0.1239 7.6487 1.0000 -0.1302 6.2919 1.0000 0.0000

2 3 180.4373 0.0000 0.0000 -0.8074 0.0000 1.0000 6.0000 0.5514

1.2490 1.0000 0.0000 1.0000 -0.0657 5.0451 0.0000 0.0000

1 4 210.9101 78.3627 55.2528 -0.2963 -0.5211 1.0000 18.9617 0.5938

2.1958 -0.1912 14.0034 1.0000 -0.1261 4.8222 1.0000 0.0000

2 4 183.1582 0.0000 0.0000 -0.7544 0.0000 1.0000 6.0000 0.3725

11.7366 1.0000 0.0000 1.0000 -0.0595 4.6177 0.0000 0.0000

4 4 80.0000 10.0000 0.0000 -0.9154 -0.4781 1.0000 17.8574 0.3198

0.6091 -0.1431 6.3117 1.0000 -0.0889 6.8565 1.0000 0.0000

1 5 0.5356 0.9614 0.0000 0.3817 -0.3000 1.0000 36.0000 0.2142

0.6116 -0.2579 6.1366 1.0000 -0.0913 6.6008 1.0000 0.0000

2 5 101.0000 0.0000 0.0000 -0.5019 -0.3000 0.0000 36.0000 0.3712

0.0705 -0.3027 15.0243 1.0000 -0.0950 6.5090 0.0000 0.0000

3 5 108.9868 10.5806 137.5564 0.8861 -0.2172 1.0000 19.1047 1.2087

0.9510 -0.1831 7.2198 1.0000 -0.1266 6.0906 1.0000 0.0000

4 5 82.5107 27.2572 137.6546 1.0000 -0.2304 1.0000 19.1688 0.4660

1.0151 -0.1596 7.8950 1.0000 -0.0909 5.5509 1.0000 0.0000

5 5 51.8235 0.0000 0.0000 0.8271 -0.3000 0.0000 16.0000 0.2670

0.2248 -0.3000 16.0000 1.0000 -0.1908 7.3978 0.0000 0.0000

3 4 184.8866 217.1354 0.0000 0.1769 -0.2406 1.0000 22.1005 0.1418

0.9091 -0.2751 8.4347 1.0000 -0.1424 6.7434 1.0000 0.0000

1 6 83.5810 9.0383 0.0000 0.2531 -0.2000 1.0000 16.0000 0.0529

1.4085 -0.1113 13.3900 1.0000 -0.1436 4.5683 1.0000 0.0000

2 6 114.7566 0.0000 0.0000 -0.8939 0.0000 1.0000 6.0000 0.1256

0.1054 1.0000 0.0000 1.0000 -0.1196 5.0815 0.0000 0.0000

3 6 105.3618 0.0000 0.0000 -0.0456 -0.2000 1.0000 16.0000 0.1870

0.7193 -0.2500 15.0000 1.0000 -0.0880 5.7169 1.0000 0.0000

6 6 91.2220 0.0000 0.0000 -0.2538 -0.2000 0.0000 16.0000 0.2688

1.4651 -0.2000 15.0000 1.0000 -0.1435 4.3908 0.0000 0.0000

5 6 56.5379 0.0000 0.0000 -0.3241 -0.2000 0.0000 16.0000 0.1607

2.6232 -0.2000 15.0000 1.0000 -0.1790 4.4051 0.0000 0.0000

4 6 79.7256 0.0000 0.0000 0.3100 -0.2000 0.0000 16.0000 0.1466

0.7435 -0.2500 25.0000 1.0000 -0.0929 5.3027 0.0000 0.0000

1 7 45.7211 -0.0200 0.0000 0.6313 -0.5000 0.0000 35.0000 0.1810

0.8439 -0.2500 11.9965 1.0000 -0.0522 4.3603 0.0000 0.0000

2 7 36.3483 0.0000 0.0000 -0.9999 0.0000 0.0000 6.0000 0.4500

2.0000 0.0000 12.0000 1.0000 -0.0697 5.0219 0.0000 0.0000

3 7 97.9383 0.0000 0.0000 -0.3751 0.3000 0.0000 6.0000 0.4328

1.3021 -0.2500 11.9965 1.0000 -0.0592 5.4275 0.0000 0.0000

4 7 91.7060 0.0000 0.0000 -0.3983 -0.5000 0.0000 25.0000 0.3470

0.0340 -0.2500 20.0000 1.0000 -0.1543 5.2475 0.0000 0.0000

7 7 33.8062 0.0000 0.0000 0.6093 0.3000 0.0000 26.0000 0.5445

0.7512 0.0000 12.0000 1.0000 -0.1361 4.4921 0.0000 0.0000

1 8 180.3526 50.0000 0.0000 -0.1860 -0.4591 1.0000 37.7369 0.2590

0.2807 -0.2047 10.2887 1.0000 -0.0641 5.9561 1.0000 0.0000

2 8 165.3660 0.0000 0.0000 -0.2658 -0.3000 1.0000 25.0000 0.3019

6.1522 0.0000 0.0000 1.0000 -0.0933 5.4815 1.0000 0.0000

3 8 236.5417 65.2243 0.0000 -0.4987 -0.2500 1.0000 25.0000 1.0000

0.9994 -0.2342 17.4842 1.0000 -0.1262 5.8863 1.0000 0.0000

4 8 0.0000 0.0000 0.0000 0.9000 -0.2500 1.0000 25.0000 0.5201

1.0000 -0.1488 10.0786 1.0000 -0.1647 6.3839 1.0000 0.0000

8 8 85.8601 0.0000 0.0000 1.0000 -0.2500 1.0000 25.0000 0.7894

0.8860 -0.2000 25.0000 1.0000 -0.0820 8.6292 1.0000 0.0000

1 9 185.5699 0.0000 0.0000 -0.6826 -0.5000 1.0000 35.0000 0.8994

4.7452 -0.2500 15.0000 1.0000 -0.1214 4.1366 1.0000 0.0000

2 9 265.0555 0.0000 0.0000 -0.6839 -0.2000 0.0000 16.0000 0.4242

49.7545 -0.2000 15.0000 1.0000 -0.0758 6.0344 0.0000 0.0000

3 9 112.9712 0.0000 0.0000 -0.4782 -0.5000 1.0000 45.0000 0.5707

1.4279 -0.2500 15.0000 1.0000 -0.0200 5.5659 1.0000 0.0000

4 9 208.5797 0.0000 0.0000 -0.9477 -0.5000 1.0000 45.0000 1.1437

1.4579 -0.2500 15.0000 1.0000 -0.1925 4.8604 1.0000 0.0000

7 9 100.5404 0.0000 0.0000 -0.5542 -0.5000 0.0000 45.0000 0.2773

3.4090 -0.2500 15.0000 1.0000 -0.0786 4.3125 0.0000 0.0000

8 9 150.6978 0.0000 0.0000 0.1373 -0.1418 1.0000 13.1260 0.3916

0.2867 -0.1310 10.7257 1.0000 -0.1182 6.8737 1.0000 0.0000

9 9 75.6988 100.5231 0.0000 0.8760 -0.3500 1.0000 25.0000 1.5575

0.0200 -0.2500 15.0000 1.0000 -0.1183 5.1151 1.0000 0.0000

1 10 0.0000 0.0000 0.0000 0.2500 -0.5000 1.0000 45.0000 0.6000

0.4000 -0.2500 15.0000 1.0000 -0.1000 10.0000 1.0000 0.0000

2 10 0.0000 0.0000 0.0000 0.2500 -0.5000 1.0000 45.0000 0.6000

0.4000 -0.2500 15.0000 1.0000 -0.1000 10.0000 1.0000 0.0000

3 10 0.0000 0.0000 0.0000 0.2500 -0.5000 1.0000 45.0000 0.6000

0.4000 -0.2500 15.0000 1.0000 -0.1000 10.0000 1.0000 0.0000

7 10 0.0000 0.0000 0.0000 0.2500 -0.5000 1.0000 45.0000 0.6000

0.4000 -0.2500 15.0000 1.0000 -0.1000 10.0000 1.0000 0.0000

9 10 153.5200 0.0000 0.0000 0.3010 -0.5000 1.0000 50.0000 0.1025

0.4150 -0.5000 15.0000 1.0000 -0.0723 5.3872 1.0000 0.0000

10 10 0.0000 0.0000 0.0000 0.2500 -0.5000 1.0000 45.0000 0.6000

0.4000 -0.2500 15.0000 1.0000 -0.1000 10.0000 1.0000 0.0000

1 11 230.6480 83.4300 132.3859 -0.7257 -0.2709 1.0000 29.9009 0.8275

3.9750 -0.3406 7.9815 1.0000 -0.1620 6.6589 1.0000 0.0000

2 11 161.1063 0.0000 0.0000 -0.1387 0.0000 1.0000 6.0000 0.7276

0.6127 1.0000 0.0000 1.0000 -0.0395 7.2218 0.0000 0.0000

3 11 85.9770 90.1220 0.0000 -0.1794 -0.4462 1.0000 34.9336 0.2655

2.1655 -0.2173 7.0137 1.0000 -0.2489 4.7873 1.0000 0.0000

4 11 83.8728 158.4962 10.6453 0.5906 -0.2136 1.0000 42.7701 0.3124

1.2522 -0.3679 5.1968 1.0000 -0.1670 4.3060 1.0000 0.0000

7 11 59.9489 0.0000 0.0000 -0.8676 -0.5000 0.0000 25.0000 0.3001

0.5172 -0.2500 20.0000 1.0000 -0.1067 4.4438 0.0000 0.0000

9 11 219.0435 0.0000 0.0000 -0.7851 -0.5000 1.0000 45.0000 0.4632

4.7945 -0.2500 15.0000 1.0000 -0.1655 4.9080 1.0000 0.0000

11 11 134.6492 66.2329 149.2707 -0.7228 -0.1000 1.0000 19.0850 1.0000

0.6060 -0.2050 9.7308 1.0000 -0.1791 5.8008 1.0000 0.0000

36 ! Nr of off-diagonal terms. at1;at2;Dij;RvdW;alfa;ro(sigma);r

1 2 0.1219 1.4000 9.8442 1.1203 -1.0000 -1.0000

2 3 0.0344 1.6800 10.3247 0.9013 -1.0000 -1.0000

1 3 0.1887 1.6622 10.4165 1.3097 1.1366 1.1191

1 4 0.3656 1.6119 10.7207 1.6790 1.3635 -1.0000

2 4 0.1017 1.7755 9.6088 1.3696 -1.0000 -1.0000

1 5 0.1495 2.0794 12.2376 0.0100 1.4060 -1.0000

2 5 0.1361 1.5875 11.9875 1.4900 -1.0000 -1.0000

3 5 0.2011 2.0377 10.4646 1.6025 1.4785 1.6595

4 5 0.2161 1.8729 9.9069 2.0896 1.6848 -1.0000

3 4 0.1822 1.8309 11.4304 1.4943 1.2907 -1.0000

1 6 0.0800 1.7085 10.0895 1.5504 1.4005 -1.0000

2 6 0.0366 1.7306 11.1019 1.2270 -1.0000 -1.0000

3 6 0.0500 1.8000 11.6139 1.4652 -1.0000 -1.0000

4 6 0.1664 1.7078 11.8610 1.7692 -1.0000 -1.0000

5 6 0.3188 2.0391 11.1208 2.3703 -1.0000 -1.0000

1 7 0.1117 1.6762 12.4579 1.6944 1.0000 1.0000

2 7 0.1165 1.5761 10.6684 1.4759 -1.0000 -1.0000

3 7 0.3585 1.4416 10.8424 1.5535 1.0000 1.0000

4 7 0.2040 2.0900 10.0100 1.8583 -1.0000 -1.0000

1 8 0.0956 1.7010 11.7436 1.3003 1.1889 -1.0000

2 8 0.0472 1.4236 11.8887 1.1333 -1.0000 -1.0000

3 8 0.0907 2.3192 9.8579 1.3103 1.2629 -1.0000

4 8 0.1000 2.0000 10.0000 -1.0000 -1.0000 -1.0000

1 9 0.0556 1.6739 11.0578 1.2427 -1.0000 -1.0000

2 9 0.0752 1.9606 9.9948 1.0331 -1.0000 -1.0000

3 9 0.1348 1.7631 10.2798 1.2347 -1.0000 -1.0000

4 9 0.3409 1.6930 10.6301 1.4936 -1.0000 -1.0000

7 9 0.1664 1.9808 9.7107 1.6587 -1.0000 -1.0000

8 9 0.0830 1.7419 10.8641 1.3592 -1.0000 -1.0000

9 10 0.1211 1.7575 9.6653 1.3555 -1.0000 -1.0000

1 11 0.1147 1.6475 10.4840 1.5441 1.0839 1.0216

2 11 0.0480 2.3000 9.0050 1.0156 -1.0000 -1.0000

3 11 0.0557 1.9804 10.1404 1.2503 1.1170 1.0125

4 11 0.2413 1.7461 9.9357 1.5934 1.4658 1.0220

7 11 0.1726 1.5073 9.4702 1.5174 -1.0000 -1.0000

9 11 0.1658 1.7458 10.5176 1.4246 -1.0000 -1.0000

133 ! Nr of angles. at1;at2;at3;Thetao,o;p(val1);p(val2);p(coa1);

3 4 3 62.5000 9.9619 3.2919 -1.0365 0.4516 0.0000 1.3077

1 4 3 75.1000 16.4780 5.0545 0.0000 0.7250 0.0000 1.0384

4 1 9 70.1233 23.4636 4.9273 0.0000 3.3034 0.0000 3.3671

9 1 9 75.1477 27.4433 4.9794 0.0000 3.4269 0.0000 3.2642

3 4 11 65.9219 42.8194 4.5719 0.0000 2.2424 0.0000 1.6980

4 11 4 58.7095 26.3208 1.0000 0.0000 1.0764 0.0000 3.3900

7 1 7 71.1401 7.4370 3.8886 0.0000 2.5612 0.0000 1.1416

7 3 7 95.7136 2.2375 4.0707 0.0000 2.7428 0.0000 2.1527

7 4 7 75.3830 5.3933 4.6274 0.0000 2.1026 0.0000 1.9606

7 9 7 92.0257 1.3076 2.5528 0.0000 2.3697 0.0000 1.0624

7 11 7 60.9696 2.0706 3.5501 0.0000 2.3388 0.0000 1.1372

1 1 1 67.2326 22.0695 1.6286 0.0000 1.7959 15.4141 1.8089

1 1 3 49.5561 7.3771 4.9568 0.0000 0.7533 15.9906 1.0010

3 1 3 124.5890 37.6977 2.5435 -24.3902 1.9329 -42.9758 2.0894

1 3 1 74.3994 44.7500 0.7982 0.0000 3.0000 0.0000 1.0528

1 3 3 115.5971 37.6251 2.0051 0.0000 0.7993 67.0264 2.9777

3 3 3 80.7324 30.4554 0.9953 0.0000 1.6310 50.0000 1.0783

3 3 4 150.2283 45.0000 5.9341 0.0000 2.7893 0.0000 1.0782

3 4 4 65.0000 45.8401 2.9557 0.0000 2.0952 0.0000 1.0323

1 1 4 68.9986 31.4057 4.8471 0.1463 0.1076 0.0000 2.5166

1 4 1 30.7500 4.5000 7.0000 0.1463 1.4452 0.0000 2.8788

1 4 4 84.4685 5.2103 6.6033 0.1463 0.0905 0.0000 2.9037

4 4 4 70.3671 5.7180 7.0000 0.0000 0.3683 0.0000 2.4869

1 1 2 65.2527 14.3185 6.2977 0.0000 0.5645 0.0000 1.1530

2 1 2 70.0840 25.3540 3.4508 0.0000 0.0050 0.0000 3.0000

1 2 2 0.0000 0.0000 6.0000 0.0000 0.0000 0.0000 1.0400

1 2 1 0.0000 3.4110 7.7350 0.0000 0.0000 0.0000 1.0400

2 2 2 0.0000 27.9213 5.8635 0.0000 0.0000 0.0000 1.0400

2 1 3 65.0000 14.2057 4.8649 0.0000 0.3504 0.0000 1.7185

1 3 2 71.5018 21.7062 0.4735 0.0000 0.5186 0.0000 1.1793

2 3 3 84.9468 23.3540 1.5057 0.0000 2.6374 0.0000 1.3023

2 3 2 77.0645 10.4737 1.2895 0.0000 0.9924 0.0000 1.1043

1 2 3 0.0000 25.0000 3.0000 0.0000 1.0000 0.0000 1.0400

3 2 3 0.0000 0.0148 6.0000 0.0000 0.0000 0.0000 1.0400

2 2 3 0.0000 9.7025 6.0000 0.0000 0.0000 0.0000 1.0400

1 2 4 0.0000 0.0019 6.0000 0.0000 0.0000 0.0000 1.0400

2 1 4 30.9196 11.3010 0.5535 0.0000 0.0050 0.0000 1.9267

1 4 2 100.0000 14.2598 4.2424 0.0000 0.0050 0.0000 3.0000

2 4 2 92.1229 42.8350 0.6163 0.0000 1.0235 0.0000 1.0010

2 4 4 70.9476 9.9024 0.6923 0.0000 0.2031 0.0000 2.9811

2 3 5 88.3222 7.1767 2.4747 0.0000 0.6219 0.0000 3.1507

2 4 5 90.0000 20.3126 0.7222 0.0000 0.6873 0.0000 2.2146

2 5 5 57.6230 6.3083 5.0722 0.0000 0.6873 0.0000 1.5510

2 5 4 54.6337 8.6317 6.9912 0.0000 1.6873 0.0000 2.8674

2 5 2 76.2482 11.2841 7.6230 0.0000 0.9375 0.0000 1.0586

2 3 4 45.6742 13.4413 1.5725 0.0000 0.7737 0.0000 2.6616

4 2 4 0.0000 7.5000 2.0000 0.0000 0.0000 0.0000 1.0400

4 2 5 0.0000 7.5000 2.0000 0.0000 0.0000 0.0000 1.0400

5 2 5 0.0000 7.5000 2.0000 0.0000 0.0000 0.0000 1.0400

2 5 3 70.0000 12.0000 4.0000 0.0000 1.0000 0.0000 1.2500

3 2 5 0.0000 15.0000 2.0000 0.0000 0.0000 0.0000 1.0500

2 1 9 85.4658 0.0100 1.9807 0.0000 1.4400 0.0000 2.9068

2 1 11 62.2465 35.4859 2.1648 0.0000 0.0110 0.0000 2.4442

2 3 11 81.0695 20.0000 2.0285 0.0000 0.1218 0.0000 1.4477

1 11 2 76.9847 49.2262 0.9407 0.0000 0.0300 0.0000 2.6196

2 11 3 85.4080 40.0000 1.7549 0.0000 0.0222 0.0000 1.0774

2 11 11 83.5658 40.0000 1.3540 0.0000 0.0222 0.0000 2.6397

2 11 2 58.0387 1.1862 3.9770 0.0000 0.0222 0.0000 1.0000

3 5 3 80.0647 49.0226 1.1861 0.7271 0.1000 0.0000 1.5321

5 3 5 16.5418 38.3796 0.5347 0.0000 0.1000 0.0000 2.3535

3 3 5 34.0844 11.5602 1.5428 0.0000 0.4319 0.0000 1.0500

3 5 5 6.0985 0.0302 0.1000 0.0000 0.6142 0.0000 1.7575

1 3 5 76.5850 8.7797 0.8099 0.0000 2.5889 0.0000 1.0500

4 5 4 66.1778 17.0744 4.2862 0.0984 1.4056 0.0000 1.7545

5 4 5 35.4696 10.5159 5.6990 0.0000 3.9985 0.0000 1.3642

4 4 5 90.0000 32.0246 1.1683 0.0000 3.9500 0.0000 1.3617

4 5 5 41.9144 0.5409 7.1700 0.0000 3.4295 0.0000 3.2326

3 5 4 75.0000 25.0000 2.0000 0.0984 1.0000 0.0000 1.5000

4 3 5 35.0000 12.5000 1.5000 0.0000 0.5000 0.0000 1.0500

3 4 5 90.0000 30.0000 1.2500 0.0000 3.0000 0.0000 1.3000

1 6 1 62.5000 16.6806 0.7981 0.0000 0.9630 0.0000 1.0711

1 1 6 87.6241 12.6504 1.8145 0.0000 0.6154 0.0000 1.5298

6 1 6 100.0000 40.4895 1.6455 0.0000 0.0100 0.0000 1.7667

1 6 6 5.0994 3.1824 0.7016 0.0000 0.7465 0.0000 2.2665

3 6 3 28.9047 27.3847 2.5790 0.0000 0.1078 0.0000 2.4145

3 3 6 90.0000 39.1857 4.8200 0.0000 0.9067 0.0000 1.9533

6 3 6 51.5671 2.9451 0.6657 0.0000 1.6341 0.0000 1.9057

3 6 6 56.7026 3.2665 4.3063 0.0000 0.6729 0.0000 2.7490

2 6 2 106.3969 30.0000 0.9614 0.0000 1.9664 0.0000 2.2693

2 2 6 0.0000 26.3327 4.6867 0.0000 0.8177 0.0000 1.0404

6 2 6 0.0000 60.0000 1.8471 0.0000 0.6331 0.0000 1.8931

2 6 6 30.3748 1.0000 4.8528 0.0000 0.1019 0.0000 3.1660

2 6 6 180.0000 -27.2489 8.3752 0.0000 0.8112 0.0000 1.0004

1 6 2 97.5742 10.9373 2.5200 0.0000 1.8558 0.0000 1.0000

1 2 6 0.0000 0.2811 1.1741 0.0000 0.9136 0.0000 3.8138

2 1 6 84.0006 45.0000 0.6271 0.0000 3.0000 0.0000 1.0000

2 3 6 28.4774 12.0885 3.2396 0.5000 0.0778 0.0000 1.6733

1 6 3 70.0000 25.0000 1.0000 0.0000 1.0000 0.0000 1.2500

1 3 6 70.0000 25.0000 1.0000 0.0000 1.0000 0.0000 1.2500

3 1 6 70.0000 25.0000 1.0000 0.0000 1.0000 0.0000 1.2500

3 2 6 0.0000 1.0000 1.3402 0.5000 0.0500 0.0000 1.5379

4 6 4 2.7962 7.1073 0.5589 0.0000 0.0554 0.0000 1.1473

6 4 6 92.9945 26.8345 0.9189 0.0000 0.0100 0.0000 1.4683

4 6 6 48.7356 9.9227 0.1206 0.0000 0.0893 0.0000 1.1108

4 4 6 64.5223 7.2562 5.2298 0.0000 0.5459 0.0000 1.0400

2 4 6 83.4937 16.7605 0.8242 0.5000 0.5409 0.0000 1.1378

4 2 6 0.0000 10.0000 1.0000 0.5000 0.2500 0.0000 1.5000

5 4 6 61.8263 20.8696 0.2450 0.0000 0.7429 0.0000 1.0400

4 5 6 60.0000 1.0000 1.0000 0.0000 1.0000 0.0000 1.2500

4 6 5 60.0000 1.0000 1.0000 0.0000 1.0000 0.0000 1.2500

5 3 6 44.9106 2.7940 0.5834 0.0000 0.9597 0.0000 1.3151

3 5 6 60.0000 1.0000 1.0000 0.0000 1.0000 0.0000 1.2500

3 6 5 60.0000 1.0000 1.0000 0.0000 1.0000 0.0000 1.2500

2 8 2 50.0000 26.9005 1.7315 0.0000 0.1848 0.0000 1.0400

2 8 8 55.2500 36.5272 6.0000 0.0000 0.4281 0.0000 2.1149

2 2 8 0.0000 10.4651 0.1000 0.0000 0.0000 0.0000 3.0000

2 3 8 75.9746 10.9523 0.8687 0.0000 1.8256 0.0000 2.9875

2 8 3 65.0000 40.0000 6.0000 0.0000 0.1000 0.0000 3.0000

3 8 3 50.4947 12.1095 3.5926 0.0000 3.0000 35.0000 1.0400

8 3 8 90.0000 40.0000 4.7885 0.0000 2.7146 0.0000 1.0400

3 2 8 52.0162 2.5267 0.3146 0.0000 2.2070 0.0000 2.9111

3 3 8 90.0000 27.7492 6.0000 0.0000 0.1870 0.0000 1.0400

8 2 8 0.5000 3.4405 0.9580 0.0000 0.8031 0.0000 1.0000

8 8 8 60.9386 12.9033 7.8607 0.0000 1.7515 0.0000 2.2405

3 8 8 70.7224 5.3644 3.4424 0.0000 0.8219 0.0000 2.8000

1 1 8 30.0491 23.9749 3.2341 0.0000 1.0000 0.0000 1.0000

1 8 1 80.6555 40.0000 5.6273 0.0000 1.0000 0.0000 3.7089

1 8 8 70.5217 39.3118 7.9958 0.0000 1.0000 0.0000 1.0000

8 1 8 47.0626 4.5590 5.6859 0.0000 1.0000 0.0000 1.4685

1 8 3 75.0000 30.0000 2.0000 0.0000 1.0000 0.0000 2.0000

1 8 2 65.0000 35.0000 4.0000 0.0000 0.5000 0.0000 2.0000

9 8 9 65.0386 28.8263 2.2480 0.0000 1.1021 0.0000 1.0400

8 9 9 70.0000 28.7353 1.2918 0.0000 1.0913 0.0000 1.0400

8 8 9 70.0000 25.0000 2.5000 0.0000 1.0000 0.0000 1.0400

9 10 9 92.7358 25.0000 2.5000 0.0000 1.1355 0.0000 1.1514

1 1 11 77.4923 42.7218 4.3622 0.0000 3.2077 50.0000 2.7926

11 1 11 74.8600 52.6870 2.0030 0.0000 3.3760 0.0000 3.0686

1 11 1 58.2601 47.8710 1.8642 0.0000 0.9406 0.0000 1.4461

1 4 11 74.8583 48.5512 6.9669 0.0000 3.4134 50.0000 1.1985

11 11 11 0.0000 27.9213 5.8635 0.0000 0.0000 0.0000 1.0400

1 4 7 130.8936 8.6656 6.9828 0.0000 3.4977 50.0000 1.5070

4 1 7 130.8936 8.6656 6.9828 0.0000 3.4977 50.0000 1.5070

4 7 4 71.3704 41.6040 1.7245 0.0000 0.1500 0.0000 1.6385

71 ! Nr of torsions. at1;at2;at3;at4;;V1;V2;V3;p(tor1);p(cot1);n

1 1 1 1 -0.2775 10.1210 0.2025 -4.6886 -2.1309 0.0000 0.0000

1 1 1 3 -0.7098 22.2951 0.0060 -2.5000 -2.1688 0.0000 0.0000

3 1 1 3 -0.0528 6.8150 0.7498 -5.0913 -1.0000 0.0000 0.0000

1 1 3 1 2.0007 25.5641 -0.0608 -2.6456 -1.1766 0.0000 0.0000

1 1 3 3 -0.0179 5.0603 -0.1894 -2.5000 -2.0399 0.0000 0.0000

3 1 3 1 -2.5000 76.0427 -0.0141 -3.7586 -2.9000 0.0000 0.0000

3 1 3 3 -2.5000 66.3525 0.3986 -3.0293 -3.0000 0.0000 0.0000

1 3 3 1 2.5000 -0.5332 1.0000 -3.5096 -2.9000 0.0000 0.0000

1 3 3 3 0.0531 -17.3983 1.0000 -2.5000 -2.1584 0.0000 0.0000

3 3 3 3 -2.5000 -25.0000 1.0000 -2.5000 -1.0000 0.0000 0.0000

0 1 1 0 -0.0098 51.9046 0.2435 -4.5500 -1.9721 0.0000 0.0000

0 3 3 0 0.5611 25.0350 1.0000 -5.3564 -0.9918 0.0000 0.0000

4 4 4 4 1.8397 -10.0688 -0.4387 -2.4875 -0.0258 0.0000 0.0000

3 4 4 4 0.2500 90.0000 0.5000 -6.0000 0.0000 0.0000 0.0000

3 4 4 3 0.2247 90.0000 0.6100 -6.0339 0.0320 0.0000 0.0000

1 4 4 1 0.0490 49.6276 0.0785 -7.5561 0.0128 0.0000 0.0000

4 4 4 7 0.0000 2.0000 0.0100 -9.0000 0.0000 0.0000 0.0000

7 4 4 7 0.0263 22.1907 0.0085 -5.3761 -0.0153 0.0000 0.0000

1 1 1 9 0.0016 19.4325 -0.2470 -4.3189 -1.8976 0.0000 0.0000

9 1 1 9 0.0100 37.2385 0.5751 -5.1000 -1.9746 0.0000 0.0000

1 1 3 7 0.0100 49.0000 0.2500 -3.8312 -0.0971 0.0000 0.0000

3 1 3 7 -0.0049 48.3830 0.2500 -3.9625 -0.0201 0.0000 0.0000

0 1 11 0 -0.7705 47.5626 0.7704 -4.3365 -3.0000 0.0000 0.0000

3 1 11 1 0.0000 90.0000 -0.2000 -2.5000 -2.0000 0.0000 0.0000

3 4 11 4 1.9416 39.7453 -1.0000 -6.4779 -2.2386 0.0000 0.0000

1 4 11 4 1.8981 39.5179 -0.9868 -6.6438 -2.2836 0.0000 0.0000

1 4 11 7 1.8670 39.7586 -0.9292 -6.5627 -2.4025 0.0000 0.0000

9 1 4 11 -0.3544 85.7240 0.3880 -3.1316 -0.1007 0.0000 0.0000

9 1 4 3 -0.2837 86.3003 0.4231 -2.6099 -0.1107 0.0000 0.0000

0 4 11 0 2.0000 90.0000 -0.7545 -9.1181 -2.0510 0.0000 0.0000

1 1 1 2 -0.2500 31.2596 0.1709 -4.6391 -1.9002 0.0000 0.0000

2 1 1 2 -0.1770 30.0252 0.4340 -5.0019 -2.0697 0.0000 0.0000

2 1 1 3 -0.3568 22.6472 0.6045 -4.0088 -1.0000 0.0000 0.0000

1 1 3 2 -1.1953 42.1545 -1.0000 -8.0821 -1.0000 0.0000 0.0000

2 1 3 1 -0.9284 34.3952 0.7285 -2.5440 -2.4641 0.0000 0.0000

2 1 3 2 -2.5000 79.6980 1.0000 -3.5697 -2.7501 0.0000 0.0000

2 1 3 3 -0.5583 80.0000 1.0000 -4.4000 -3.0000 0.0000 0.0000

3 1 3 2 0.0345 78.9586 -0.6810 -4.1777 -3.0000 0.0000 0.0000

1 3 3 2 -2.5000 3.3219 0.7180 -5.2021 -2.9330 0.0000 0.0000

2 3 3 2 2.2500 -6.2288 1.0000 -2.6189 -1.0000 0.0000 0.0000

2 3 3 3 0.4723 -12.4144 -1.0000 -2.5000 -1.0000 0.0000 0.0000

3 3 3 3 -2.5000 -25.0000 1.0000 -2.5000 -1.0000 0.0000 0.0000

0 1 2 0 0.0000 0.0000 0.0000 0.0000 0.0000 0.0000 0.0000

0 2 2 0 0.0000 0.0000 0.0000 0.0000 0.0000 0.0000 0.0000

0 2 3 0 0.0000 0.1000 0.0200 -2.5415 0.0000 0.0000 0.0000

0 2 4 0 0.0000 0.0000 0.0000 0.0000 0.0000 0.0000 0.0000

2 1 3 5 2.1344 29.9850 0.3398 -3.1459 -2.1000 0.0000 0.0000

1 1 3 5 0.4573 10.0000 1.0000 -7.3632 -2.1000 0.0000 0.0000

2 3 5 3 0.3709 10.0000 0.9625 -9.0000 -1.0000 0.0000 0.0000

2 3 4 3 2.5000 2.5000 0.2237 -10.0000 0.0000 0.0000 0.0000

1 4 4 2 0.0000 50.0000 0.0000 -8.0000 0.0000 0.0000 0.0000

2 4 4 2 0.0000 50.0000 0.0000 -8.0000 0.0000 0.0000 0.0000

1 1 1 6 0.0000 5.0000 0.4000 -6.0000 0.0000 0.0000 0.0000

6 1 1 6 0.0000 44.3024 0.4000 -4.0000 0.0000 0.0000 0.0000

2 1 1 6 0.0000 21.7038 0.0100 -4.0000 0.0000 0.0000 0.0000

2 1 6 1 0.0000 5.2500 0.0100 -6.0000 0.0000 0.0000 0.0000

1 1 6 1 0.0000 5.1676 0.0100 -5.9539 0.0000 0.0000 0.0000

1 1 6 2 0.0000 5.1676 0.0100 -5.9539 0.0000 0.0000 0.0000

6 3 3 6 0.0509 30.0000 0.5000 -4.0000 0.0000 0.0000 0.0000

0 8 8 0 0.0000 42.3911 -0.3192 -4.3105 0.0000 0.0000 0.0000

0 3 8 0 -2.0000 48.7726 -0.5000 -2.5000 0.0000 0.0000 0.0000

8 3 3 8 2.0000 75.0000 0.3000 -5.0000 0.0000 0.0000 0.0000

0 1 8 0 0.0000 30.0000 -0.1000 -5.0000 0.0000 0.0000 0.0000

1 1 1 8 0.0000 2.0000 0.0000 -6.0000 0.0000 0.0000 0.0000

8 1 1 8 0.0000 2.0000 0.0000 -6.0000 0.0000 0.0000 0.0000

2 1 3 7 0.0000 50.0000 0.2000 -4.0000 0.0000 0.0000 0.0000

1 11 1 2 -0.4607 73.2745 0.8057 -6.5581 -2.7334 0.0000 0.0000

11 1 1 2 -0.2828 45.1749 0.1156 -6.2783 -2.5478 0.0000 0.0000

1 1 1 11 -0.5541 76.7989 0.7541 -8.0269 -1.9959 0.0000 0.0000

1 1 11 1 -0.4431 75.1291 -0.7926 -1.6886 -2.8003 0.0000 0.0000

11 1 11 1 -2.2533 83.2815 -0.4856 -7.9986 -1.0007 0.0000 0.0000

7 ! Nr of hydrogen bonds. at1;at2;at3;r(hb);p(hb1);p(hb2);p(hb3

3 2 3 1.9682 -4.4628 1.7976 3.0000

3 2 4 2.5000 -1.0000 1.7976 3.0000

4 2 3 2.5000 -1.0000 1.7976 3.0000

4 2 4 1.5000 -2.0000 1.7976 3.0000

3 2 11 2.0000 -2.5000 1.7976 3.0000

11 2 3 2.0000 -2.5000 1.7976 3.0000

11 2 11 2.0000 -2.5000 1.7976 3.0000

- The structure file of a Frenkel defected Li_2S structure "Fr_infdil_786_Vtzp_Ah.data" can be found in SI_input_data.zip folder;

- the structure file of a Li_2S bulk with 3% Li vacancies ("vac_Li_p3b_Li2S.data") and other density of vacancy defects (i.e. 0.3%, 2%) can be found in SI_input_data.zip folder;

- the Input file of each NVT run for various density of defects: 0.3%, 2%, 3% at various temperature (300, 600, 700 and 800 K) and for defective Frenkel case can be found

in SI_input_data.zip folder both in presence and in absence of an applied electric field.

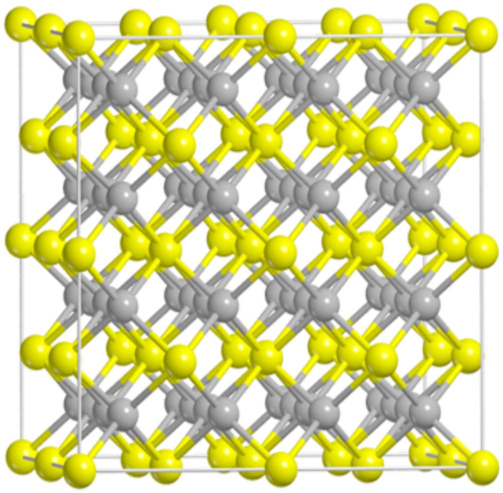

Figure S1: $2 \times 2 \times 2$ supercell (96 atoms) of Li_2S conventional cubic bulk cell in the $Fm\bar{3}m$ symmetry group.

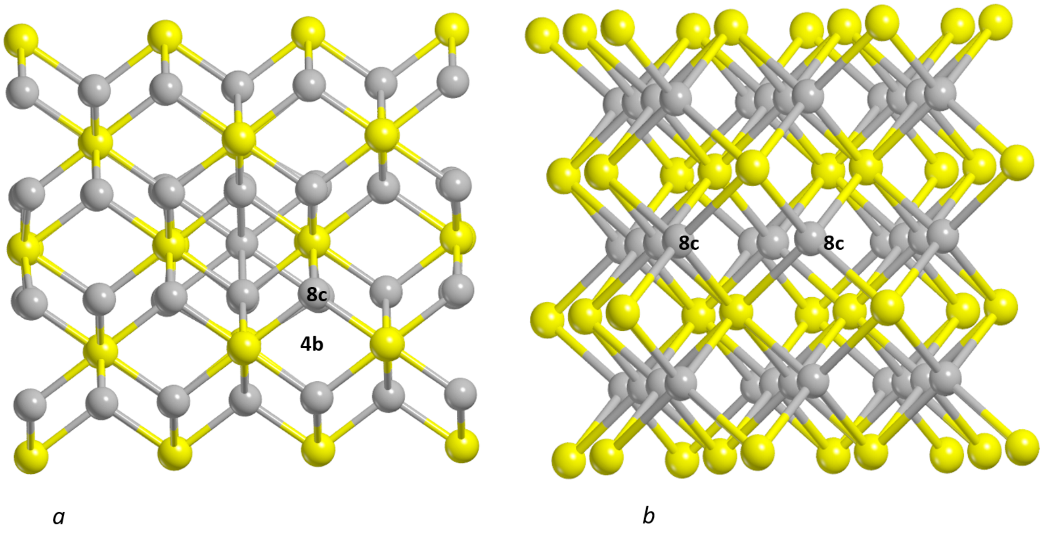

Figure S2: Sites 4b and 8c in $2 \times 2 \times 2$ supercell (96 atoms) of Li_2S conventional cubic bulk cell in the $Fm\bar{3}m$ symmetry group (different views **a)**and **b)**). Li-ions occupy these sites during migration process according to both vacancy and Frenkel mechanisms as described in the main text.
